# Nature-Based Interventions and Mind–Body Interventions: Saving Public Health Costs Whilst Increasing Life Satisfaction and Happiness

**DOI:** 10.3390/ijerph17217769

**Published:** 2020-10-23

**Authors:** Jules Pretty, Jo Barton

**Affiliations:** 1 School of Life Sciences, University of Essex, Colchester CO4 3SQ, UK; 2 School of Sport, Rehabilitation and Exercise Sciences, University of Essex, Colchester CO4 3SQ, UK; jobarton@essex.ac.uk

**Keywords:** nature-based interventions, mind–body interventions, life satisfaction, happiness, green social prescribing

## Abstract

A number of countries have begun to adopt prevention pays policies and practices to reduce pressure on health and social care systems. Most affluent countries have seen substantial increases in the incidence and costs of non-communicable diseases. The interest in social models for health has led to the growth in use of social prescribing and psychological therapies. At the same time, there has been growth in application of a variety of nature-based and mind–body interventions (NBIs and MBIs) aimed at improving health and longevity. We assess four NBI/MBI programmes (woodland therapy, therapeutic horticulture, ecotherapy/green care, and tai chi) on life satisfaction/happiness and costs of use of public services. These interventions produce rises in life satisfaction/happiness of 1.00 pts to 7.29 (*n* = 644; *p* < 0.001) (for courses or participation >50 h). These increases are greater than many positive life events (e.g., marriage or a new child); few countries or cities see +1 pt increases over a decade. The net present economic benefits per person from reduced public service use are £830–£31,520 (after 1 year) and £6450–£11,980 (after 10 years). We conclude that NBIs and MBIs can play a role in helping to reduce the costs on health systems, while increasing the well-being of participants.

## 1. Introduction

### 1.1. Prevent to Save 

Recent decades have seen substantial improvements in a wide range of health indicators in populations of affluent countries, resulting in better infant survival, more effective treatments of cancers and infectious diseases, and greater longevity [1,2,3]. At the same time, however, a suite of non-communicable diseases (NCDs) have increased in incidence across whole populations, the most costly comprising mental ill health, dementias, obesity and type 2 diabetes, loneliness and cardiovascular disease (including strokes) [4,5,6,7]. NCDs worldwide now constitute more than 50% of annual deaths. As a result of both unhealthy longevity and expansions in incidence of these NCDs, public health and social care systems have been facing growing financial pressure on services [8,9].

The UK Chief Medical Officer [10] proposed that these health costs arising from ways of living required a “new canon for prevention.” With an ageing population, growing numbers of people with long-term health conditions (LTCs), cost inflation, and pressures on revenue funding, the health systems in affluent countries need to find ways to invest in prevention to slow the pipeline of people requiring primary and secondary treatment. This concern for prevention is, of course, not new: in 1736, Benjamin Franklin wrote that “an ounce of prevention is worth a pound of cure” (referring specifically to fire safety), and since then, the axiom has been widely used in reference to health systems. What has changed recently in affluent countries has been the scale of costs and the pressing need for changes to policy and investment priorities. In the UK, the prevention pays policy was expanded in 2018 while setting out ambitions for system-wide changes by 2040 [8,11], with a raised focus on health as an asset that needed protecting: “prevention creates the right conditions for good health and wellbeing - helping everyone to live well for longer” [12]. There is a need, in short, to focus on “prevention to slow the growth in demands on the National Health Service (NHS)”, making it sustainable for future generations [12].

The annual direct costs of NCDs is now a substantial personal and economic burden. Poor diet, physical inactivity, fractured social structures, lack of access to green spaces and consumption of alcohol and recreational/prescription drugs, combined with some genetic factors, have resulted in a sharp increase in costs of NCDs [3]. In the UK, the cost to the NHS of six NCDs has been calculated to be some £62 billion per year, with the full cost to the whole economy at £160 billion [6,13]. The treatment costs of these conditions thus represent 35% of the annual revenue (running) costs of the NHS (£177 billion, 2019–2020). It has been estimated that 15 M people in the UK (23% of the population) suffer from long-term health conditions (LTCs) [8], and these people make up 50% of GP (general practice) appointments and 70% of hospital inpatient days. Many LTCs are mental ill health related, often termed the diseases of despair.

Successful public health responses at the population level remain rare. Exceptions include mass vaccination programmes, the removal of lead from petrol (1980s–1990s), the growing legal and cultural restrictions on tobacco (resulting in a 20 year drop in incidence from 30% of adults to 17% by 2018), the increasing numbers of participants in Park Runs (from 2004 up to 350,000 participants weekly). The Covid-19 self-isolation and lockdown programmes common to many countries in 2020 represent the largest ever cost-prevention programmes imposed by public policy.

Here we assess the potential value of social models for health, in which changed behaviours and choices of individuals could create the conditions by which they stay happier and healthier for longer in life. Our focus is on nature-based and mind–body interventions (NBIs and MBIs), and how adoption could lead to reduced cost pressure on health and social care and other public services.

### 1.2. Social Models of Health

In the past two decades, it has become clear that the structure of the social, ecological and economic environments in which people live and work is an important shaper of health and well-being outcomes. This has been called a “social model of health” [8]. It has also become apparent that growth in average national income, as measured by GDP per capita, does not result in increases in life satisfaction/happiness above a GDP per capita threshold of between $10 and 20,000 [6,14,15,16,17]. This Easterlin paradox [18,19] suggests that (i) life satisfaction/happiness (LS/H) may be influenced by non-material behaviours and choices in the economy, and that (ii) GDP is a poor measure of well-being and happiness once basic needs have been met [17,20,21,22].

In most affluent countries, including the UK, GDP per capita has increased several fold over half a century, yet LS/H at the population level has remained persistently stable [14,15,17]. Individuals have more stuff (material goods), but these have not made each person on average happier [15,23]. It is clear that uncosted activities are influencing LS/H, and so not included in the measures and assumptions about the economic success of countries; it is also clear that overcosted activities are included in GDP where they clearly result in negative health outcomes (e.g., air pollution or road traffic accidents). Recent evidence has also shown that net well-being (life satisfaction/happiness) across populations is reduced when inequality grows [24,25], and where there is breakdown of social structures and support [26], lack of access to natural and green spaces [27,28], and consumption of unhealthy foods [29].

One example of a modern non-communicable disease affected by the breakdown of social structures is loneliness [30,31,32]: it has an acute negative effect on health (equivalent to smoking 15 cigarettes daily), and increases annual GP visits by 1.8-fold and annual accident and emergency (A&E) visits by 1.6-fold [8]. Loneliness and social isolation increase mortality by 25–30% over 7 years [33]. At the same time, it is clear that the socially connected live longer and are happier [34], and countries with higher levels of trust in other people are happier [17,22]. Volunteers who contribute to the well-being of others and to the quality of lived environments tend to have healthier lifestyles, lower incidence of mental ill health, and live longer [17,35,36,37].

The presence of green and accessible natural space is also known to be important to incidence of NCDs. Green space close to residence reduces mortality [38], reduces levels of stress hormones [39], increases levels of physical activity [40], changes dietary decisions and habits [29,41], and affects longevity of the elderly [42]. Regular exercise and rich social networks add 5.4 years to life [43], walkers live longer [44] and healthy plant-based diets play a key role in reducing mortality [29,41,45]. It is also clear that life satisfaction and happiness plays a direct role in longevity [17,22]. The happiest third of the population aged >50 have a 10% chance of dying over the next 9 years, the middle third a 20% chance, and the least happy a 30% chance [46].

A range of prospective life-course studies and large-scale evaluations (e.g., Caerphilly, Dunedin, Maudsley/Cambridge cohorts, Milwaukee nuns, Harvard ageing, and New England centenarians) have further shown how combinations of choices about behaviours and consumption patterns directly affect health and well-being over many decades [4,42,47,48,49,50,51], how the structure of natural and social environments shape health, and how the value of early interventions demonstrated by studies of children whose adult health and well-being outcomes are improved when exposed to activities in natural places (playgrounds, gardens, and woodlands) [52,53,54].

### 1.3. Social Prescribing and Psychological Therapy Models

The UK health system has developed two important “prevention pays” intervention models in recent years. Social prescribing (SP) was developed by GP surgeries to offer alternatives to medical intervention (NASP, 2019), and the Improving Access to Psychological Therapies (IAPT) programme provides novel interventions for patients with depression and anxiety [55,56]. These have been called non-clinical community interventions (NCCIs) [57], and are particularly focused on people and patients with NCDs. Both types of intervention now provide valuable data on costs and beneficial outcomes for individuals and health systems.

Social prescribing is a relatively recent term applied to the systems of referral used by GP primary health care operators. It has been defined as “supporting people via social prescribing link workers to make community connections and discover new opportunities, building on individual strengths and preferences, to improve health and well-being” [58]. The longest serving SP operation is at Bromley-by-Bow (25 years), and there are exemplars acknowledged in Brighton, Doncaster, Dudley, Frome, Leeds, London (City and Hackney, Croydon, Tower Hamlets), Newcastle, Rotherham, and Yorkshire and Humber [57,58,59,60,61,62,63,64]. The most effective SP programmes employ Link Advisers as guides to patients, interviewing and assessing needs, and then ensuring they can take advantage of the community-based options available with good signposting. These fall into distinct categories: (i) for advice and knowledge (e.g., on benefits or housing); (ii) for skills development (e.g., computing, food and cooking); (iii) for activities in social groups (e.g., befriending and self-help groups, dance, art, or crafts); and (iv) for activities with therapeutic design, especially nature-based (e.g., walking for health or woodland therapies) and formal counselling. Most well-developed SP operations have >50 options for onwards patient referral; some have more than 200 options (e.g., Bromley-by-Bow).

The National Academy for Social Prescribing (NASP) was established by the NHS in 2019, and is seeking to expand SP as a new social movement [58]. The NASP notes that SP is part of an aim to prescribe bespoke personalized medical and social interventions, but also note that health improvements may be “long slow journeys” for many patients. The term green prescribing has been used to refer to the nature-based options available to SP operations [65,66].

The Improving Access to Psychological Therapies (IAPT) programme was established in 2008, and a range of talking and cognitive therapies are now recommended by National Institute for Health and Care Excellence (NICE). These include cognitive behavioural therapy (CBT), interpersonal psychotherapy, couples therapy, counselling, psychodynamic therapy and mindfulness-based cognitive therapy [55,56]. These are offered to address a wide range of mental health conditions such as depression, general anxiety disorder, post-traumatic stress disorder (PTSD), panic disorders, obsessive-compulsive disorder, and body dysmorphic disorder. Some 560,000–600,000 patients are treated each year, with full recovery rates of 40–50% recorded, and some 67% of people making “reliable improvements” [12,55,67,68]. These efficacy rates are better than most reported uses of antidepressants [69,70]. IAPT made 1.6 M referrals for talking therapies in 2019—1.09 M started courses, and 52% completed courses (583,000), with a range of 7–20 sessions attended per person [67].

Evaluations of SP programmes have measured reductions in GP visits (by 12% per person over 6 months, by 28% over 12 months); falls in A&E visits (by 24% over 1 year); reductions in secondary care appointment (down by 35% over 1 year). For each £1 invested in SP, benefits are £1.43 (3 months); £2.30 (1 year); £1.98 (5 years); and reduced costs of the order of £250–500 per person per year. The Healthy London partnership for Social Prescribing has noted that London could have saved £110 M over 2013–2016 if SP had been widely available and used.

### 1.4. Nature-Based Interventions (NBIs) and Mind–Body Interventions (MBIs)

It is now well established that exposure to nature has positive health and well-being benefits. There has been a rapid expansion in evidence, showing in a wide variety of contexts that green exercise (physical activity in the presence of nature) improves health and well-being [13,17,71,72]. Every social group benefits: all ages, genders, ethnicities and social classes respond positively to green exercise. All natural environments have been found to be beneficial: from urban parks to biodiversity rich, from small local to large landscapes, from domestic gardens to the farmed and wild landscapes [40,73]. The phrase *dose of nature* was coined to indicate that exposure to green exercise is analogous to a medicinal dose, improving mental health and well-being in the short and long term [74,75,76].

The natural environment is now understood to provide vital health services as well as other ecosystem services [77,78]. In 2019, the UK’s 25 Year Environment Plan published by the government [79] set out six priorities, one of which was to “connect people with environments to increase health and well-being”, and substantial focus has been put on making use of green spaces and natural habitats to improve the health of whole populations. A wide range of nature-based interventions (NBIs) have been developed across conservation, wildlife and community organisations and charities [72,80,81,82,83,84,85]. These natural and social therapies have been deployed for groups under mental stress, at-risk children and young people, refugees, probationers, and dementia sufferers [65]. Fields in Trust [86] show that parks and green spaces provide some £34 billion of health and well-being benefits annually, saving the NHS >£100 m per year; the benefits equate to £974 per person per year to replace life satisfaction gained from local parks and green spaces.

Notable programmes for NBIs include (i) forest and woodland schools [87,88]; (ii) wilderness and adventure therapies [89,90]; (iii) ecotherapies and green care [91,92]; (iv) social farms and gardens [65]; (v) dementia prevention and treatment [93,94] walking for health (Walking the Way to Health set up 500 walking groups for some 170,000 people [80]).

At the same time, a wide range of mind–body interventions and practices (MBIs) have become popular, usually delivered by individual trainers or community groups. Many millions of people practice yoga, tai chi and other forms of mindfulness, either individually or with groups, and often in natural environments (tai chi/qigong—65 M daily practitioners in China, 7.5 M in the USA). The evidence base on health impacts is substantial. There have been >500 studies of the health benefits of tai chi, mindfulness, yoga and qigong on tens of thousands of participants [95,96,97,98,99,100,101,102,103,104,105,106]. Following a mix of periods of practice (short to medium courses, short- to long-term adoption), these have resulted in improvements to bone density, cardiopulmonary function, reduced body mass, reduced anxiety, and improved quality of life.

In both NBIs and MBIs, the key behaviour (and later habit when automated without thought) that affects life satisfaction and happiness is the practice of attentiveness and immersion [13,107,108]. This can produce altered states of mind [109,110,111], also called flow [112], resulting in a downregulation or quieting of the mental chatter that is a common source of anxiety and mental ill health [113]. We have proposed a simple brain metaphor, with levels representing different stages of mammal–hominid evolution; see [13,114,115,116]. The brain stem (bottom brain) is the oldest and contains survival functions: it is fast responding, involuntary, automatic, impulsive, driven by emotions, and is the executor for habits and routines. The top-brain cortex is the most recent, having expanded in size rapidly during the later stages of hominid evolution: it is slower, voluntary, able to learn and plan, and contains centres for social abilities of empathy and language. The top brain is calming, drives the parasympathetic nervous system (PNS), and is characterised by rest and digest. The bottom brain is spiked into action by the amygdala, the sentinel for threats, drives the sympathetic nervous system (SNS), and is characterised by fight and flight.

There is, though, no off switch for the thinking part of the pre-frontal cortex. This default mode can only be downregulated by choices we make with the body. Attentiveness and immersion (A&I), also called the relaxation response (RR) [117,118], have been shown to induce transient hypofrontality in the brain’s default mode network (DMN) [119,120,121,122,123]. The relaxation response can also build grey matter, particularly in the hippocampus and thus increasing memory; this induction of the RR by MBIs moderates the expression of 3800 genes [118], changing the internal responsiveness to stressors. Systematic reviews have shown the reduced expression (downregulation) of genes involved in inflammatory reactions, reducing risk of inflammation and related diseases [124,125]. In this way, NBIs and MBIs reduce the effects of stress, increase well-being and reduce anxiety, depression and pain.

### 1.5. Theory of Change for Interventions

Two questions are central to the success of all non-clinical community interventions (NCCIs: SP, IAPT, NBIs and MBIs): (i) does the intervention result in measurable health and well-being benefits, and (ii) does the intervention induce the adoption of repeated habits that result in long-term positive outcomes and prevention of costs? Most interventions focus more strongly on the former (core design components of the intervention), and many pay no attention to the nature of habit formation. A common aim of all interventions should be to shift personal behaviours and choices towards easily repeatable daily actions that lead to sustained improvements in health and well-being. An effective dose of nature, for example, was shown in a meta-analysis [74] to be highly effective in improving mental health and well-being in the first five minutes of exposure in a green space. However, this alone will not produce permanent changes in neural structure unless it provokes behaviours and habits that individuals can easily repeat [113,126].

Habits are fixed action patterns (FAPs), producing automated behaviour or thought patterns without intention. Triggers may be external or internal, and result in behaviours proceeding to completion once initiated. Well-practised behaviours recur because performance has become automated, thus saving the brain from using resources [127]. Habit strengths are higher if repeated, so that they do become involuntary [128], and then hard to change [129,130]. Adherence is a key hope for the application of all NCCIs: an immediate benefit from a short-lived activity can increase life satisfaction, but permanent changes to brain and body will only occur from repeated practice [131,132,133].

As indicated above, planning and learning occurs in the pre-frontal cortex (PFC) of the brain, and as routines are automated so they are sent downwards to the mid and lower brains, and tend to remain fixed unless brought back up for amendment [13]. As a routine is habituated, so the basal ganglia take over from the PFC, allowing us to pay less attention (and the brain to use less energy). All habits require many hours of practice, but once learned no longer require active attention (for example, walking gait and language). Many of the modern conditions of ill health result from behaviours gradually adopted over time and are thus hard to change, including eating and drinking habits, smoking, sedentary lifestyles, reduced direct contacts with family and community, reduced contact with green places, and increased use of pharmaceutical solutions to ill health.

In the increasingly typical modern life course, many people cede territory, inch by inch, to automated behaviours and habits that often bring discontent, unhappiness and ill health. We pay less attention to eating well, forget friendship groups, and become less active. Yet new habits and behaviours need forceful and sometimes fierce action, not least because there are economic and social structures that many people cannot avoid (e.g., design of lived environments and housing) or are actively encouraged by companies and advertising to adopt (e.g., consumption of unhealthy foods and drink or gambling). This is the social model of health—the contexts of lived circumstances shape health outcomes, often without people appreciating the influences.

There are thus many economic and social influences that are beyond the capabilities and responsibilities of both individuals and delivery organisations. It is not a matter of choice that individuals are in some way unwell before referral, nor that they may be out of work or lacking safe housing options, not whether they have fallen into health-damaging and socially costly habits (such as with alcohol or recreational drugs). It is well established that living within 1 km of urban green space improves well-being and healthy longevity [42], yet many people may face physical and cultural barriers that prevent access to such green space. It is well known that five fruit and vegetable portions eaten daily improves health in the long term [134,135], yet 20% of the UK adult population consumes zero portions daily [136]. Corporate advertising encourages adults and children to consume unhealthy foods, which are often cheaper and more available than healthy options owing to the nature of the food system [137]. Ambient air pollution affects health, yet individuals are mostly unable to avoid pollution and its effects in the short term (unless by moving location or effectively lobbying for policy change). It is within these contexts that nature- and social-based interventions must act.

Experimental research has shown that it takes 28 to 84 days to form a habit, depending on the time devoted each day [126,133]. We have thus suggested a heuristic (rule of thumb) of 50 h of practice to hardwire a new habit (equivalent to 50 days at one hour per day, or 100 days (3 months) at half an hour per day) [13]. A period of intense cognitive engagement occurs while learning the habit (high activity in the PFC), and is then followed by the shift of the new routines to the basal ganglia. The habit is then automated without thought. However, changing ingrained habits is hard: we have to force the body to do something different, as it will not choose voluntarily to do so.

These habits may be developmental (learning to walk as a toddler, handwriting, learning to drive a car) or deliberative (creating new behaviours for well-being, such as diets or giving up smoking). The 50 h benchmark represents the period of active cognitive engagement with the new activity. Many habit-forming programmes (such as diets) do not persist for long enough to form new neural structures, and are thus doomed to fail in the long term. The cravings of habits are hard to overcome [113]: having made the effort to learn and adopt, the brain does not wish to devote valuable resources to dispose of old habits and develop new ones.

Thus a theory of change for effective programmes of social and green interventions (by NBIs and MBIs) centres on the need for the following key components, following the COM-B model of behaviour change [138,139]:

(i) *Opportunity*: A logic of adherence—there need to be incentives and motivations for individuals to complete the course or programme.
(ii) *Motivation*: The ability of individuals to incorporate the new behaviours, habits and activities into their lives beyond the course/programme in order to sustain health and well-being benefits over the long term, resulting in reductions in demand for public health and other public services (e.g., education support, criminal justice actions and support).
(iii) *Capability*: A programme design that is intended to produce long-term and positive changes in individuals.

## 2. Materials and Methods

### 2.1. Four NBI and MBI Programmes of Intervention

We have selected four NBI and MBI programmes where data were comparable to assess the impacts of intervention, and use evaluations of both SP and IAPT programmes [12,58,59,61] and measures of life satisfaction/happiness [21,22] to provide external benchmarks.

#### 2.1.1. Green Light Trust (NBI)

The Green Light Trust (GLT) is a charity providing natural and social interventions for a wide range of vulnerable groups in the east of England. The GLT supports adults and young people facing challenges in their lives by using nature as the medium for engagement. The Trust supports individuals facing a broad range of challenges including mental health issues, substance misuse, special educational needs, veterans, women and families in a refuge, those in probation services and those most vulnerable and marginalised in or on the periphery of the education system. The GLT delivers woodland therapy to 1500 adults and children each year, and seeks to provide new life-course pathways for the vulnerable and hard to reach in society. Before the advent of Covid-19, this work was predominantly delivered in a natural woodland setting, where social isolation could be addressed whilst increasing confidence, self-esteem and progression in life. The key components of woodland therapy are learning about natural history, craft activities, preparing and cooking food, led discussions and walking in woodlands. This delivery method has been adapted and support is continuing for the adults and young people. In 2020, the GLT developed a blended online delivery model, calling its land-based programmes called *Earth*, and the online delivery and support called *Air*.

#### 2.1.2. Ecominds (NBI)

The five-year Ecominds project was run by the mental health charity Mind (2007–2012), and benefitted 12,000 people in 130 ecotherapy/green care projects [91,140]. These comprised nature-based interventions in a variety of natural settings. These interventions included some formal therapy (e.g., counselling sessions, cognitive behavioural therapy, or psychotherapy), as well as informal therapy of the programme of NBI activities. The projects were effective in raising mental well-being of participants (all adults), in enhancing social inclusion, vital to the recovery of those living with mental health problems, in both increasing connections with nature, enabling participants to benefit further from the associated health and well-being benefits, in improving well-being and social inclusion, and in leading to the development of healthier lifestyles and environmentally friendly living. Many projects have since continued to provide services for people with mental health challenges: green care components, for example, have been developed into the care farming delivered by 250 operations to 9000 vulnerable people each year [65].

#### 2.1.3. Trust Links Growing Together (NBI)

The Growing Together project was established in Westcliff in 2000 by independent local charity Trust Links to address the mental health needs of people living in Southend-on-Sea. This social and therapeutic gardening project supports the recovery and well-being of adults experiencing mental health problems, people with learning disabilities, and people living with other disabilities. The aim was to complement existing clinical services and help people in their recovery journey as they progressed to independence. Trust Links’ vision is to provide nurturing and supportive environments to develop strong, resilient and healthy people and communities. Since the flagship site in Westcliff opened in 2000, the Growing Together model has been replicated in three coastal regions of Essex, UK (Shoeburyness in 2012, Thundersley in 2015, Rochford in 2016). Members typically attend 1 to 2 days per week (50–100 times per year) to access therapeutic horticulture as well as a range of peer-support and vocational activities including music, art, creative writing, yoga relaxation, cooking and crafts. Accredited vocational training is provided for work-related qualifications. Trust Links also supports members to work towards employment through a Job Club and one-to-one employment support. Across the country, there are some 1000 horticultural therapy projects with 21,000 annual adult and child users [65].

#### 2.1.4. Living Movement Tai Chi (MBI)

Tai chi is an ancient form of exercise arranged into set moves and sequences. It is defined as a mind–body exercise rooted in multiple Asian traditions, including martial arts, traditional Chinese medicine, and philosophy. Tai chi training integrates slow and intentional movements with breathing and cognitive skills [97]. Programmes are generally open to participants, and do not rely on formal referrals, for example from social prescribing operations (though informal recommendations may have been made). This tai chi is thus institutionally similar to many yoga or mindfulness programmes—open to all, but less likely to attract people with more severe therapeutic needs. The assessed tai chi MBI programme is delivered by Living Movement in Devon, and led by tai chi master Angus Clark [141]. There are many forms of tai chi and qigong: this programme is based on the short form developed by Cheng Man-ch’ing [142,143]. It has been estimated that 12% of the UK population engage in yoga each week, 7% in various forms of meditation and 21% in prayer daily [17,144].

### 2.2. Assumptions

We include analysis of four datasets (*n* = 642 people): from Green Light Trust (*n* = 32), Trust Links Growing Together (*n* = 328), Ecominds green care interventions (*n* = 154), and a tai chi programme (*n* = 128). We present findings on the costs saved, and benchmark the life satisfaction/happiness benefits created with other life events and equivalent income values in the UK. Cohorts were evaluated for the GLT data (2019–2020), TL-Growing Together over the 2017–2020 period; Ecominds from 2013; tai chi over the 2016–2018 period. Data were gathered by staff of the NBI operations (trained by the University of Essex), and thus under local ethics rules and guidance.

A range of impact data were gathered for the four programmes. We focus here on the key question relating to life satisfaction/happiness adopted by the ONS in 2013, and on recorded data on the use of public health and other services by clients and participants. The positivity question has been widely benchmarked in national panel surveys [145,146], and in national well-being and happiness comparisons [17,21,22]. Some evaluations of SP and NBI programmes have measured the impact on GP appointments and hospital admissions, and the costs saved have been calculated using a range of different methods [65,87,140].

Here we make the following assumptions about the creation of a benefits register:

- We use the guidance and methods set out by the government’s Treasury Green Book [147] for benefits appraisal. The unit costs saved for public services are drawn from the New Economy Manchester cost–benefit analysis spreadsheets and unit costs database (v 2.0 updated April 2019), and draw upon costs in Pretty et al. [6]. We use best practice recommended in the Treasury Green Book by calculating cost savings over 1 and 10 years. We use a discount rate of 3.5% (costs saved today are worth more than those in the future), and a standard 2% GDP deflator (to take account of inflation). This permits creation of a net present social value (NPSV) for interventions.
- We use the norm of “reliable improvement” as an outcome measure [56,67]. Evaluations of Improving Access to Psychological Therapies (IAPT) programmes show that 40–50% of individuals make complete recoveries, and some 63–67% make reliable improvements over the long term. This suggests that some 30% of individuals do not benefit from the designed interventions. We thus differentiate where data exist between participants who make “reliable improvements” to their well-being and those who receive no benefit.
- A number of biases may be inherent in these analyses [147]. There may be an optimism bias of observers and deliverers of programmes that unintentionally inflates beneficial outcomes. There may be an unmeasured counterfactual of natural recovery: some improvements to individuals would have occurred regardless of the intervention, as would some adverse outcomes. Displacement may also occur: where one benefit achieved may be at the cost to another service. Regional variations in economic costs of programmes and service delivery are not included, nor can account be taken of cultural norms of behaviours and habits that may vary across social classes and at different locations in the UK. We also do not make adjustments for gender differences in uptake of programmes (SP typically shows a gender bias in uptake of 60% women, 40% men) or of outcomes (NBI programmes have found no differences in outcomes according to gender). Finally, no specific adjustments have been made for the substantial social, economic and cultural changes occurring since the worldwide outbreak of the Covid-19 virus.
- Economic evaluations of SP and NBI programmes have often included in their benefits register the costs saved from reductions in benefits payments (on the assumption that individuals have returned to work), and the increase tax contributions from such employment. The evidence is also compelling that these types of programmes can result in measurable improvements to self-esteem and mood, improved sleep patterns, and changed habits that would lead to greater happy longevity. Here we focus on costs saved from three types of public services: health and social care, police and criminal justice, and education. We also analyse the marginal improvements in life satisfaction and happiness, and indicate the substantial changes required in society to achieve similar benefits. We conclude that economic benefits measured by these interventions are minima, as not all changes have been monetised.

## 3. Results

### 3.1. Impacts on Changes to Life Satisfaction/Happiness

**Figure 1:**
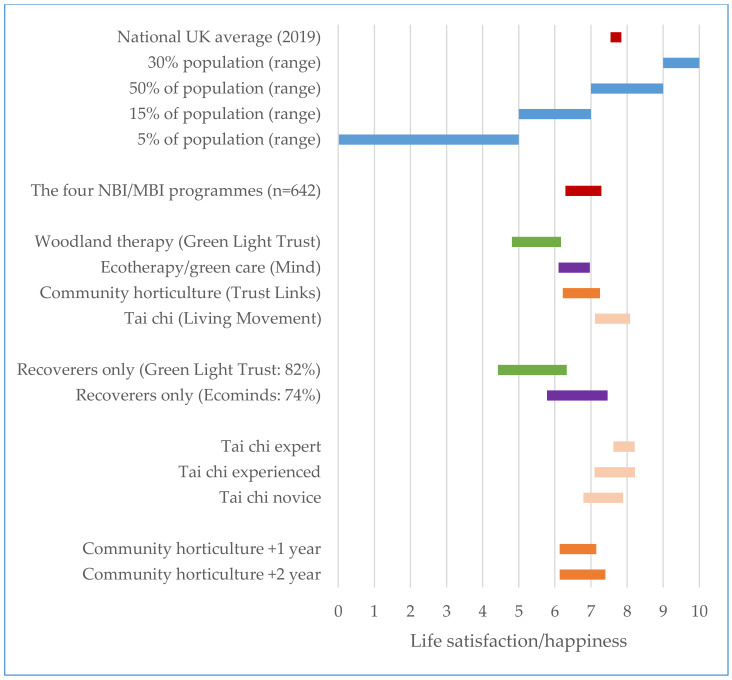
Margins of improvement of life satisfaction/happiness following four nature-based and mind and body interventions.

There is a growing understanding of the science of happiness [17,21,22,146]. The UK government’s Office for National Statistics (ONS) has been measuring well-being since 2013, and the World Happiness Reports measure annual changes in happiness across >150 countries and >150 cities. We have been able to assess the impacts on LS/H of the four NBI and MBI programmes. Figure 1 shows the marginal changes in LS/H, benchmarked against the UK average (7.69 for 2019), and the proportions of the UK population recording scores between 0 and 4.99 (5%), 5.00 and 6.99 (15%), 7.00 and 8.99 (50%) and 9.00 and 10.00 (30%) [17,148].

Table 1 and Figure 1 contain the mean changes in LS/H across all programmes (+1.00 pts on the 1–10 scale; *p* < 0.001). The woodland therapy of the Green Light Trust increased LS/H by +1.36; the ecotherapy/green care of Ecominds by +0.87; the therapeutic horticulture of Trust Links by +1.03; and the tai chi of Living Movement by +0.97 (all *p* < 0.001). The published literature shows that it is generally hard and/or costly to make changes of +1 pt at the population level [21,22,146]. Existing levels are stubborn, and positive and negative changes as a result of significant life events are usually <1.0 (such marriage and birth of a child; divorce and loss of job: [145,146]. The What Matters Course run by Action for Happiness (8 weeks duration) trained 1500 people by mid-2019, and produces a 1 pt average increase in LS/H after two months on the course [17].

We have segmented the subpopulations of non- and negative responders for the GLT (23.1% of participants), Trust Links (10.6%) and Ecominds (26.2%). These are the participants who have not benefitted from the intervention or who have become worse. There were no non-responders in the tai chi sample. The proportion of non- or negative responders is lower than in SP and IAPT programmes (recorded as approximately 35%). For those who responded positively to the NBIs, the marginal improvements in LS/H rose to +1.91 for the GLT, +1.60 for Trust Links, and +1.68 for Ecominds (all *p* < 0.001). The negative responders saw falls in LS/H of between −1.00 and −2.32.

We were able to segment the tai chi data according to prior length of practice using the 50 h habit benchmark to indicate novices (0–50 h), a 10-fold increase for experienced (50–500 h), and a further 10-fold increase for experts (500–5000+). The starting LS/H was highest for experts and lowest for novices; the marginal increases were highest for novices and experienced practitioners. The end point of 8.2 after intervention practice for experienced and expert was still below the top 30% of the national population (scoring 9–0). The Trust Links data also show a greater increase in LS/H for practitioners of horticulture over 2 years (+1.27) than over 0–12 months (+1.02).

A further important factor is the LS/H characteristic of the populations of patients, participants or clients of the NBIs and MBIs. The UK mean score for LS/H (2019) was 7.69, with 20% of the population recording scores of 0.00–6.99. A score in LS/H of <3.0 is equated with misery. All four programmes started with mean levels of LS/H below the national average. The GLT population started at 4.81, indicating a referred population in the bottom 5% of the national population. Ecominds and Trust links started at 6.11–6.43, in the bottom 20% of population. The tai chi self-referred population begins closest to the national average (6.79–7.62) and ends above the population average at 8.2 for experienced and expert practitioners.

### 3.2. Impacts on Costs Saved

Benefits can be presented in two ways: (i) the ratio of social benefits to cost of delivery; and (ii) the net present value of the benefit created (cost saved), at 1 year and 10 year time periods [147]. No published analyses of SP, IAPT and NBIs-MBIs have used the 10 year point recommended by the UK Treasury Green Book, and there is no accepted standard evaluation point after intervention or course, though most are conducted at end of course or within a year of completion.

We calculate the economic benefits of NBI/MBI programmes using three measures: (a) reduced costs on public health and other services, (b) reduced health costs arising from reduced loneliness, and (c) economic benefits created from LS/H improvements in income equivalents. We do not have data on changes to employment status post-interventions, so do not calculate potential reductions in benefit claims and consequent increases in tax contribution to the public purse, as has been calculated elsewhere [140], nor do we have data on avoided prescription use/costs or prevented deployment of community nurses (e.g., psychiatric) [87].

(a) Some evaluations of social prescribing programmes have recorded outcomes on service use, again though over variable time periods after intervention. The ranges for improvements after one year are 15–25% reductions in GP appointments, 20–25% reductions in A&E appointments, and 35–50% reductions in secondary treatments in hospitals [58]. We only have data on the use of public services from the GLT NBI (Table 2). These show changes in the use of GP appointments, A&E visits to hospitals, and recorded engagements with police and criminal justice systems.

Using the Treasury Green Book (2019) and Manchester New Economy (2019) data, the savings for the GLT NBI have been calculated at the year 1 and year 10 time points (Table 3).

(b) We assume that the NBI programmes reduced loneliness and social isolation, as there are explicit aims to increase both nature and social connectedness. Loneliness increases annual GP visits by 1.8-fold and annual A&E visits by 1.6-fold [8]. We assume that these are reduced to the levels found in the GLT cohort. This results in additional Y1 savings of £714 and Y10 NPSV (savings) of £5317 (Table 3).
(c) A third approach is to benchmark the changes in income that would be needed to achieve the equivalent improvements in LS/H observed in these NBI/MBI programmes [87,146,149]. Collins [150] has calculated that a 1 pt increase in LS/H produces higher economic benefits to individuals starting at lower LS/H: for those on the median income of £23 k, an increase of 1 pt from 4 to 5 is equal to +£13,100 of income; for those on 5 to 6, £9230 of income; for those on 6 to 7, £7140 of income, and for those on 7 to 8, £5920 of income. We assume that these benefits accrue only once, so are counted only in year 1. Taking the mean LS/H increase of +0.97 pt, after one year, the NBI of the Green Light Trust thus adds £12,097 of value to each individual; Ecominds and Trust Links add £6550, and the Living Movement tai chi £5430. The 10 year benefits are smaller, as both discount rates and inflation are added each year (Table 3).

A wide range of social benefit–cost ratios have been recorded in the literature for social prescribing and NBI programmes, again with no consistent time point for measurement. For SP programmes, benefit–cost ratios range from £1.25 to 1.50 for each £1 invested (at 1 year) and £2.00 for each £1 (at 5 years) [58]. For NBIs, such as care farms, green gyms, city farms, garden and horticulture projects, rates of return are typically 1.4:1 to 4.0:1, though some appear to rise to 6.9:1 to 10:1 [65,87].

Table 4 summarises the costs of intervention and delivery in SP, IAPT and NBI programmes: these vary per individual from £320 to £1400 [61,68,82,146]. This variation is partly explained by the length of designed intervention and specificities of content: for example, some programmes may appear to be low cost, but the hours of delivery might be too few to be effective in forming sustainable new habits and behaviours.

The ratios of benefits to cost of delivery for the Green Light Trust and Trust Links NBIs are shown in Table 5, and include the segmented returns for the subpopulation of GLT responders only. We do not have equivalent costs for Ecominds because of the wide variety of projects, nor for the Living Movement tai chi training and personal practice. The benefit–cost ratios at Y1 are 0.86–1.58 and between 6.81 and 12.47 at Y10, indicating a payback period of 14 months (for full cohort) and 7.5 months (for responders only). These social returns on investments in the GLT NBIs are favourable compared with other NBI and SP programmes (typically 2.0–5.0 to 1.0), and highly beneficial when compared with no intervention.

## 4. Discussion

The literature on life satisfaction and happiness makes a very clear observation: it is difficult to achieve movements of +1 pt on the 1–10 pt scale. In the World Happiness Reports [21,22], the maximum change across whole populations in life satisfaction/happiness (LS/H) over a decade (2005–2006 to 2016–2018) is 1.0–1.3 (5 countries), then 0.5–1.0 (30 countries) and 0–0.5 (43 countries). From 2008–2012 to 2017–2019, 10 countries >1.0, 31 between 0.5 and 1.0, and 34 countries 0–0.5. Over a decade, only 5–10 countries and no cities see increases of >1.0 pt (Table 6). In summary, it is hard to move even one point on the LS/H scale. It is clear that against these changes, the NBI/MBI programmes achieve important changes in LS/H for responding participants. As shown in Table 1, a proportion of participants are non- or negative responders (11–26%): this may be because the intervention does not suit them or is ineffective, or because external negative life events have intervened during the course.

A second approach to benchmarking the marginal changes in LS/H observed in the NBI/MBI programmes is to compare with the changes that result from significant life events (Table 7). Clark et al. [145,146] have measured the effect on LS/H of key life events, both positive (e.g., new partnership or marriage) and negative (e.g., loss of job). They find that LS/H may rise or fall before an event (in anticipation) and/or persist afterwards (with personal scarring if negative). All the effects of positive life events are less than 1.0; the effects of negative events can be to reduce LS/H scores by −0.4 to −1.3. Once again, it is clear that against the changes provoked by life events, the NBI/MBI programmes achieve important changes in life satisfaction and happiness for participants that could add to other changes and/or offset the effects of negative events and stressors.

A third consideration is the effect on equity. We indicated that the client cohorts on the four NBI/MBI programmes started with mean LS/H scores below the UK population mean (7.69). It has been noted in the literature that a 1 pt increase for those starting low is worth more (to the individuals and for society as a whole as it decreases inequity) than a 1 pt increase from a higher start (e.g., 8 or 9) [17,146]. The GLT client population starts lowest, in the lowest 5% of population, yet shows the largest increases of +1.36 to +1.82. Those for Ecominds and Growing Together started higher at 5–6 (in the bottom 20% of the population), and achieve increases of +0.87 to +1.03. The tai chi population started closer to the national average, and achieved changes of +0.97. Some of the tai chi practitioners can be assumed to have benefitted from long-term engagement with the practice, and all were self-referred, having made lifestyle choices to engage with the practice.

The natural and social interventions detailed here are thus deliberate interventions helping to reduce health inequalities [24,25,151]. They tend to be targeted more at people with low absolute health and well-being indicators (below the population average) and demonstrate that improvements can be made for individuals that result in reduced social inequality. This benefits all of society, as greater equality results in greater LS/H for all [17,22]. The World Happiness Reports [21,22] make clear that the structures of social-ecological-economic environments are a strong influence on happiness [22], and national surveys (e.g., DCMS [144]) on volunteering and social networks show the importance of social context in shaping LS/H and well-being. The World Happiness Reports [21,22] make clear that the greatest explanatory factors of differences in LS/H across countries and changes over years is prosociality, freedom to make choices, healthy life expectancy at birth, generosity of others, and low inequality. The effects of corruption and perceived dystopia are strong factors in reducing LS/H. As shown above, we have observed a mean increase as a result of the four NBIs/MBIs of +1.00 from the interventions (*n* = 642, *p* < 0.001), rising to +1.88 for the segmented population of GLT responders.

We draw attention to some limitations to this research that will point toward new directions for research on the impacts of NBIs and MBIs. The results point toward rational policy responses, but as yet no preventative policies have been adopted at a scale that can be evaluated. We have not taken account of the potential effects of differing levels of biodiversity and landscape quality type utilized by the three NBIs and one MBI. We have only evaluated one MBI (tai chi) here: there are a range of types of tai chi and qi gong practiced, and these may produce differing outcomes. Evidence of other MBIs, such as yoga, pilates and prayer, would increase the range of knowledge on cost-effective interventions. Finally, these four NBIs/MBIs combine a range of components, including physical activity, cognitive engagement, presence of nature, social connections and togetherness, and healthy food preparation and consumption. We are unable to say whether these are interacting with each other or independently contributing to happiness and life satisfaction.

However, a wide range of evidence does now show that a package of six life-course or lifestyle components increases the probability of healthy, happy and long lives. These comprise (i) access to and use of natural places and green space, (ii) regular physical activity (especially in green spaces: green exercise), (iii) healthy and mixed diets, (iv) being part of and embedded in rich social networks, (v) maintaining cognitive capability across the life course, and (vi) a life lived with a spiritual or meaning framework [17,42,50,152,153].

## 5. Conclusions

We have shown that the package of four NBIs and MBIs was effective at raising LS/H of patients and participants. The increases of 1 pt and above are hard to achieve at country and city levels (though sample sizes here are smaller), and here we find that the client population all start at LS/H below the UK population average, particularly for the three NBIs, showing that the less well and happy are able to benefit.

The economic returns have been calculated from both avoided costs on public health and other services, and from the equivalent income increases required to raise life satisfaction/happiness. The total economic returns to NBIs/MBIs are £6000–£14,000 per person after Y1, and £8600–£24,500 per person after Y10. The economic returns for prevented costs only (not counting effects on LS/H) are £800–£1500 at Y1, and £5300–£12,300 at Y10. A single cohort of 10 responders for the GLT would thus produce £22,350 of savings for public services in one year, and £173,000 of savings after 10 years.

For the GLT woodland therapy, the ratios of public benefit–private cost of delivery are 1.71–2.47 after Y1 (for costs prevented only), and rise to 15.1–15.8 when effects on life satisfaction/happiness are included. The ratios after Y10 rise to 12.9–27.1:1. For the Trust Links community horticulture, the total benefits to costs are 6.42 for Y1 and 7.61 for Y10. It is thus clear that NBIs can contribute to the “prevention pays” policy of the NHS and the government [8,10], along with the growing expansion of support for social prescribing and psychological therapies [56,58].

Some form of screening clearly occurs before the therapeutic programmes of intervention, with referring organisations making assumptions about who might benefit. We note that the proportion of non- or negative responders is smaller (10–26%) than those recorded in social prescribing and IAPT programmes (approx. 35%), but we cannot conclude that these programmes could be beneficial for all. Some participants may be affected by external events during a course, and thus show negative responses independent of the effect of the NBI.

We are also unable to say whether these NBIs and MBIs will result in adherence over a long period of time. However, programmes with at least 50 h of practice do have a greater chance of forming automated habits and behaviours capable of delivering long-term health benefits. The Ecominds green care projects were variable in length, but the Green Light Trust and Trust Links programmes are designed to have participants exceed the 50 h habit-forming norm, and the data from the Living Movement tai chi practitioners show that two-thirds were experienced or experts (>50 h practice). Some segments of data indicate a changing dose–response relationship over time, with both community horticulturalists and tai chi practitioners showing higher LS/H with increasing practice over several years.

These data suggest public policy at local and national levels should encourage investments in NBIs and MBIs, as programmes at scale will deliver substantial benefits and contribute to the aspirations of the CMO [8] to prevent cost pressures on the health and social care sectors. The government’s 25 Year Environment Plan [79] sets out the importance of natural habitats and green space for health, and further points to the contribution social prescribing can make to national health and well-being.

However, targets for participation have not yet been set by either health or environment ministries. New Zealand issued an explicit well-being budget in 2019, and Sweden has set a *Vision Zero* target for road traffic accidents, accepting that there will be individual errors, but that the national target should be zero deaths [8]. In the UK, a soft drinks levy was introduced in April 2018 [9], and was expected to remove 45 M kg of sugar per year from soft drinks. No country, though, has as yet set similarly ambitious targets for non-communicable diseases (NCDs): for example, returning levels of adult obesity to 1990 levels (from 30% to 3%), reducing diabetes and loneliness incidence (by 10-fold), reducing mental ill health from 16% of the adult population in any one year to 1.6% (10-fold reduction), or increasing fruit and vegetable consumption from 3.5 to 5 portions per day (and thus for 20% of the population, from zero to 5).

The Marmot Review [151] of health inequalities concluded that “economic growth is not the most important measure of our country’s success,” and prioritised the accumulation of the positive effects on well-being across the whole life course by building social capital, encouraging active travel, use of public transport, availability of green space and healthy eating, and promotion of nature-based interventions for health. We conclude that greater adoption of formal and informal NBIs and MBIs would increase national well-being and reduce inequalities of health. A number of effective training programmes are also available to increase happiness [17].

A greener economy that emphasises ecological public health [6,154,155,156,157] would be one in which attention is paid to the environmental and social context of the public not yet ill, and all patients and professionals engaged in treatment and care [8,10]. It might also address the most pressing global challenge of climate change driven by material consumption patterns, where world greenhouse gas (CO_2_ equivalent) emissions need to be reduced from 53 to 10 Gt annually to create a safe place for humanity [15,158,159]. This will require new habits and behaviours to be taken up by whole populations, particularly those that encourage non-material consumption over material goods, and then adherence over sustained periods [16,160,161].

The Department for Health and Social Care [9] observed that “prevention and early intervention programmes represent very good value for money” in that poor health and well-being can be improved while at the same time taking cost and pressure away from primary and secondary health and public service systems. There are high levels of satisfaction with social and green prescribing [63,66], but it also important that these programmes do not become a reason for cutting public services. We conclude that nature-based and mind–body interventions can play an important role in helping to achieve these aspirations, particularly in a post-Covid-19 world, where economic and social stresses on individuals and health systems will be higher.

## Figures and Tables

**Table 1:** Summary of impacts of NBIs and MBI on life satisfaction/happiness.

NBI/MBI	Sample Size	Start LS/H Score (Mean ± Standard Deviation)	End LS/H Score (Mean ± Standard Deviation)	Margin of Change	Significance (Bonferroni-Adjusted *t*-Test)
**All Participants**					
Woodland Therapy: Green Light Trust	32	4.81 ± 2.12	6.17 ± 1.64	+1.36	(*p* < 0.01)
Horticulture: Trust Links	328	6.22 ± 2.03	7.25 ± 1.93	+1.03	(*p* < 0.001)
Green Care: Ecominds	154	6.10 ± 2.33	6.97 ± 2.24	+0.87	(*p* < 0.001)
Tai Chi: Living Movement	128	7.11 ± 1.75	8.08 ± 1.51	+0.97	(*p* < 0.01)
Summary: All Interventions	642	6.29 ± 2.12	7.29 ± 1.99	+1.00	(*p* < 0.001)
**Segmented: Responders Only**					
Woodland Therapy: Green Light Trust	26	4.42 ± 1.68	6.33 ± 1.25	+1.91	(*p* < 0.001)
Horticulture: Trust Links	285	6.16 ± 2.08	7.76 ± 1.66	+1.60	(*p* < 0.001)
Green Care: Ecominds	122	5.78 ± 2.27	7.46 ± 1.96	+1.68	(*p* < 0.001)
**Segmented: Negative Responders Only**					
Woodland Therapy: Green Light Trust	6	6.50 ± 3.08	5.50 ± 2.86	−1.00	(*p* = 0.28)
Horticulture: Trust Links	43	6.40 ± 0.79	5.05 ± 1.30	−1.35	(*p* < 0.001)
Green Care: Ecominds	32	7.38 ± 2.11	5.06 ± 2.23	−2.32	(*p* < 0.001)
**Impact over 2 years**					Mixed ANOVA
Horticulture: Trust Links (0–12 months)	166	6.13 ± 1.92	7.15 ± 1.86	+1.02	(*p* < 0.001) ^a^
Horticulture: Trust Links (12–24 months)	60	6.13 ± 1.92	7.40 ± 2.02	+1.27	
**Impact of Prior Practice/Expertise**					Mixed ANOVA
Tai Chi—Novice (0–49 h)	53	6.79 ± 1.85	7.89 ± 1.48	+1.10	*(p* = 0.046) ^b^
Tai Chi—Experienced (50–499 h)	42	7.10 ± 1.57	8.22 ± 1.35	+1.12	(*p* < 0.001) ^c^
Tai Chi—Expert (500–8000 h)	33	7.62 ± 1.76	8.21 ± 1.75	+0.59	

^a^ Significant main effect for time (F(1,418) = 61.6, *p* < 0.001), but no significant interaction or main effect for duration of practice. Post-hoc analyses confirm that scores significantly increase over time irrespective of duration of practice. ^b^ Significant interaction effect (F(2,125) = 3.1, *p* = 0.046) and a ^c^ significant main effect for time (F(1, 125) = 101.8, *p* < 0.001), but not group. Post-hoc analysis confirms that scores significantly increase over time for each of the three groups, but there is no statistically significant difference in these changes between the groups.

**Table 2:** Changes in public health/service use (GLT cohorts) over 12 weeks.

	Changes in Number of GP Visits (over 12 Weeks of Programme Compared with Previous 12 Weeks)	Changes in Number of A&E Hospital Visits	Changes in Police Incidents
GLT—whole cohort	−2.50	−0.44	+0.03
GLT—responders only	−2.84	−0.75	−0.06

**Table 3:** Per capita economic benefits created in NBI/MBI programmes from reduced use of public services, reduced loneliness and increased life satisfaction/happiness.

Costs and Benefits	NBI/MBI	Benefits after Year 1	Benefits after Year 10
A: Costs prevented from reduced public services use (*n* = 32 or 26)	Green Light Trust		
	Full cohort	£831	£6456
	Responders only	£1521	£11,980
B: Costs prevented from reduced loneliness (*n* = 671)	Green Light Trust, Ecominds, Trust Links, Living Movement	£714	£5317
C: Benefits created from increased LS/H (income equivalent) per individual	Green Light Trust	£12,097	£7271
	Trust Links	£6550	£3963
	Ecominds	£6550	£3963
	Living Movement	£5430	£3285
Total economic benefits per person (A + B + C)	Green Light Trust		
	Full cohort	£13,642	£14,332
	Responders only	£14,332	£24,568
	Trust Links *	£7264	£9280
	Ecominds *	£7264	£9280
	Living Movement *	£6144	£8602

Note: Annual discount rates of −3.5% and inflation of −2% applied per year; Y0 = year of NB intervention; Y1 = end of first year; Y10 = end of ten years; NPSV = net present social value; years of cohort interventions and evaluation: 2016–2020. No data * on reduced GP/A&E costs from Ecominds, Trust Links and Living Movement.

**Table 4:** Costs of delivery of NBI, SP and IAPT programmes.

Programme	Length of Programme	Intervention Time for each Individual	Cost (£) per Person	Sources
Green Light Trust: Earth	12 weeks	72 h	960	Green Light Trust (pers comm)
Green Light Trust: Air	10 weeks	60 h	845	
Nature-Based Interventions (general)	Variable	24–36 h	320	[82]
Trust Links Growing Together	Open access all year	Uptake: 1–2 sessions per week (50–100 per year)	1130 per year	Trust Links (pers comm)
Social Prescribing	Variable		153	[61]
Mindfulness-Based Cognitive Therapy for Depression	8 weeks + refreshers over months 5–14	18 h + 4 refresher hours	112	[100]
IAPT—Low Intensity	7 weeks	7	493	[68]
IAPT—High Intensity	7 weeks	20	1416	
IAPT—All		7–20 sessions (x 1 hr)	680	[55,56]

**Table 5:** Ratio of public benefits–private costs of delivery.

	End Y1	End Y10
Green Light Trust (prevented public health and services costs only)		
Full cohort	1.71	12.9
Responders only	2.47	19.1
Green Light Trust (total benefits)		
Full cohort	15.1	15.8
Responders only	15.8	27.1
Trust Links (total benefits)		
All	6.42	7.61

Note: Cost of the GLT NBI = £960 per person per programme (2020); Trust Links £1130 per person per year.

**Table 6:** Number of countries and cities showing positive movements in life satisfaction/happiness over approximate 10 year periods.

Changes in Life Satisfaction/Happiness (LS/H) across Whole Populations	Number of Countries		Number of Cities
	2005–2006 to 2016–2018	2008–2012 to 2017–2019	2005 to 2013
Increases >1.0	5	10	0
0.5–0.99	30	31	22
0.0–0.49	43	34	66
Falls by −0.01 to −1.5	50	71	81

Source: WHR [21,22].

**Table 7:** Effect of life events on life satisfaction/happiness scores (data from household panel surveys: UK, Germany, and Australia).

Significant Life Event	Women/Men	Effect on LS/Happiness (on 1–10 Scale)	Changes in 5 Years after the Life Event
Partnership	Women	+0.5 to +0.7	Stable
	Men	+0.4 to +0.5	
Parenthood	Women	+0.4	Stable
	Men	+0.2	
Separation/Divorce	Women	−0.5 to −1.0	Then rising by +0.5 after 4–5 years
	Men	−1.0 to −1.3	
Widowhood	Women	−0.6 to −1.0	Then returning completely after 1 year
	Men	−0.5 to −1.0	
Loss of Employment	Women	−0.4	Then falling to −1.2 after 5 years
	Men	−0.4	

Source: Clark et al. [145,146].
